# Left-Side Contrast-Injection-Induced Pseudopathologic Vertebral Enhancement in Oncology Patients without Venous Obstruction: A Report of Two Cases

**DOI:** 10.3390/medicina60010079

**Published:** 2023-12-30

**Authors:** Kyungsoo Bae, Jin Il Moon, Kyung Nyeo Jeon

**Affiliations:** 1 Department of Radiology, Institute of Medical Science, Gyeongsang National University School of Medicine, Jinju 52727, Republic of Korea; ksbae@gnu.ac.kr (K.B.); logan63@gnu.ac.kr (J.I.M.); 2 Department of Radiology, Gyeongsang National University Changwon Hospital, Changwon 51472, Republic of Korea

**Keywords:** collateral circulation, computed tomography, contrast media, vertebra, vertebral metastasis

## Abstract

The appearance of sclerotic bone lesions in contrast-enhanced computed tomography (CT) scans is often a significant concern for the possible presence of metastatic disease, especially in individuals with a known history of cancer. Prior research has demonstrated that in cases where patients suffer from thrombosis in major veins like the superior vena cava or the brachiocephalic vein, vertebral venous congestion can create imaging patterns on CT scans that resemble sclerotic bone metastases. However, instances of such imaging findings in patients without any form of venous obstruction are not commonly reported. In this study, we present cases of pseudopathologic vertebral enhancement observed consistently following left-side contrast injections in cancer patients devoid of venous obstruction. We aim to discuss and propose a potential mechanism for this phenomenon, drawing attention to a less commonly recognized diagnostic consideration in oncological imaging.

## 1. Introduction

Computed tomography (CT) is a pivotal imaging modality for staging and follow-up in patients with various types of cancer. It plays a crucial role in the detection of vertebral metastases, which is an essential aspect of follow-up CT scans for cancer patients. This detection significantly impacts the prognosis determination and the formulation of treatment strategies. Bone metastases can manifest as lytic, sclerotic, or mixed lesions, with each type reflecting a different primary mechanism of interference with normal bone remodeling.

Pseudopathologic vertebral enhancement is a documented phenomenon that can complicate the interpretation of these scans, often simulating osteoblastic metastasis [1,2]. This type of enhancement is generally associated with paravertebral venous congestion, particularly in patients with obstructions in the superior vena cava (SVC) or brachiocephalic vein (BCV). While instances of pseudopathologic enhancement are relatively uncommon, they can be erroneously interpreted as sclerotic bone metastases in clinical settings, leading to potential misdiagnoses. It is noteworthy that vertebral venous congestion and pseudopathologic enhancement can also occur in the absence of obstructions in the SVC or BCV, further complicating the diagnostic process.

This case report presents two unique instances of pseudopathologic vertebral enhancement following left-side contrast injections in cancer patients without any venous obstructions. These cases underscore a less common but significant diagnostic challenge, emphasizing the need for the careful evaluation of vertebral enhancements in CT scans for cancer follow-up. The importance of considering all variables, including the method of contrast injection, is crucial in these assessments.

## 2. Case Report

**Figure 1:**
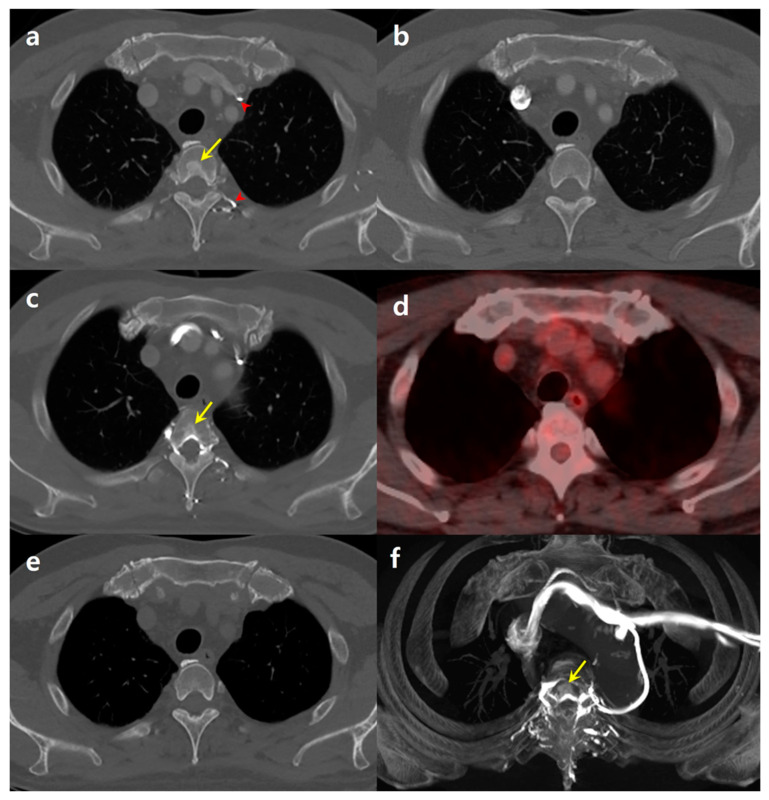
A 65-year-old male patient with a history of colon cancer surgery. (**a**) Contrast-enhanced chest computed tomography (CT) image, obtained with left-side contrast injection using bone window setting, reveals focal sclerotic area at the central portion of the T3 vertebral body (arrow). Notably, contrast arrival is observed in the left brachiocephalic vein and the left paravertebral venous plexus (arrowheads). (**b**) Chest CT obtained six months ago with right-side injection shows no evidence of a sclerotic lesion in the T3 vertebral body. (**c**) Chest CT obtained 11 months ago with left-side injection reveals the presence of focal sclerotic lesion (arrow) in the same location. (**d**) 18F-FDG PET-CT reveals no abnormalities in the same vertebra site. (**e**) A follow-up unenhanced chest CT conducted six months later from (**a**) shows no sclerotic lesion. (**f**) Maximum-intensity projection (MIP) image reconstructed from an enhanced CT reveals a focal enhancing area at T3 (arrow), with the surrounding vertebral venous plexus opacified through reflux from the left brachiocephalic vein.

A 65-year-old male, who had successfully undergone surgery for colon cancer five years earlier, was engaged in ongoing surveillance for any signs of cancer recurrence or metastasis. Throughout this period, he maintained good health without any indications of local cancer recurrence or distant metastasis. As part of his routine follow-up, the patient underwent a chest CT scan. For this procedure, contrast material was administered through his left antecubital vein. The enhanced CT images, viewed using a bone window setting, revealed an unexpected finding: a focal sclerotic lesion located centrally in the T3 vertebral body (Figure 1a). This lesion was notably absent in a CT scan taken six months earlier, where the contrast injection had been performed on the right side (Figure 1b). There was no evidence of deep venous obstruction in any part of the patient’s body.

The patient’s medical records showed that he had undergone a total of nine chest CT scans since his operation for colon cancer. Of these, five were conducted with right-side contrast injection and four with left-side injection. Intriguingly, none of the CT scans with right-side injection showed any focal sclerotic lesions in the vertebrae. However, all four chest CT scans involving left-side injection consistently revealed a sclerotic area at the same T3 location, although the extent of the sclerotic area varied slightly (Figure 1c). Further investigations using 18F-FDG PET-CT showed no abnormalities at the same vertebra location (Figure 1d). Additionally, the sclerotic lesion was not visible in unenhanced CT images (Figure 1e). Maximum-intensity projection reconstruction of the contrast-enhanced CT images revealed a focal enhancing area in the T3 body, which was attributed to contrast reflux from the left brachiocephalic vein (BCV) through the paravertebral venous plexus (Figure 1f).

**Figure 2:**
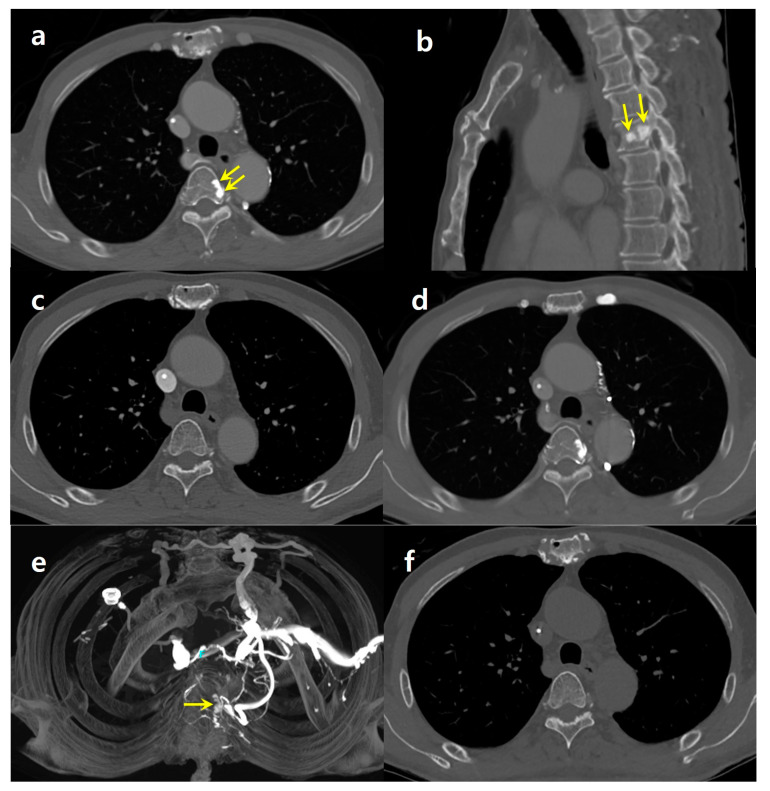
A 69-year-old male with gall bladder cancer. (**a**) An axial contrast-enhanced chest CT image with left-side contrast injection reveals a focal sclerotic area on the left side of the T4 vertebral body (arrows). A chemoport catheter is inserted in superior vena cava. (**b**) Sagittal reformatted image shows nodular sclerotic foci at T4 (arrows). (**c**) Chest CT image obtained three months ago with right-side injection does not show the sclerotic lesion. (**d**) Chest CT image obtained six months ago with left-side contrast injection shows a focal sclerotic area in the same location. (**e**) Maximum-intensity projection (MIP, d) image from enhanced CT with left-side injection demonstrates a focal enhancing area at T4 (arrow), with the surrounding vertebral venous plexus opacified through reflux from the left brachiocephalic vein. Collateral flows are also noted in both internal mammary and left superior intercostal veins. (**f**) A follow-up chest CT with contrast injection through his right arm showing no evidence of a sclerotic lesion in T4 or paravertebral venous collaterals.

A 69-year-old male patient, who had undergone a radical cholecystectomy for gallbladder cancer seven years earlier, was under continuous surveillance for potential metastasis following his surgery and subsequent adjuvant concurrent chemoradiation therapy. As part of his ongoing monitoring, he recently underwent a contrast-enhanced chest CT scan. In this particular scan, the contrast agent was administered through his left antecubital vein. The chest CT images revealed a focal sclerotic lesion at the left lateral aspect of the T4 vertebral body (Figure 2a,b). Notably, this lesion had not been visualized in the CT scan obtained three months earlier, which had involved contrast injection through his right arm (Figure 2c). The imaging studies, including CT scans, ultrasonography, and angiography performed during chemoport insertion, showed no evidence of deep venous obstruction.

Upon reviewing the patient’s imaging history at our institution, it was found that he had undergone a total of 13 chest CT scans. Interestingly, a sclerotic area was consistently observed at the same location in the CT scans where the contrast was injected from the left side (Figure 2d). However, the seven chest CT scans obtained with right-side contrast injection did not reveal any focal sclerotic lesions in the vertebrae. A maximum-intensity projection reconstruction of the contrast-enhanced CT scans demonstrated focal enhancement at the T4 vertebra, which was attributed to the flow through the paravertebral venous plexus (Figure 2e). This enhancement was not present in the unenhanced follow-up CT images (Figure 2f), indicating that the phenomenon was likely related to the distribution of the contrast agent.

## 3. Discussion

The bone is commonly affected by secondary metastatic diseases, with the spine being a particularly frequent site of bone metastasis. The vertebral venous plexus, characterized by its extensive connections, not only serves as an alternative pathway for venous return but also facilitates the spread of malignant cells. The discovery of new areas of increased density within the vertebrae on follow-up CT scans can lead to concerns about sclerotic metastases in patients receiving oncological treatment. There have been several documented instances of pseudopathologic vertebral enhancement in patients with thoracic vein obstructions, complicating the interpretation of bone lesions on CT scans in cancer patients [3,4]. This phenomenon is typically caused by an increased retrograde flow of contrast agents into the paravertebral venous plexus, resulting in localized enhancement of the vertebral bodies. Such occurrences have been previously described in patients with venous obstructions, including thrombosis in the SVC or BCV [1,2,3,4,5]. However, reports of this phenomenon in patients without venous obstruction are rare.

In our study, we report two cases of pseudopathologic enhancement in thoracic vertebrae where there were no thoracic venous obstructions. Intriguingly, this phenomenon occurred exclusively when contrast agents were administered via the patients’ left arms. These cases present a unique diagnostic challenge, underscoring the importance of the side of contrast injection in CT imaging and its potential influence on the interpretation of radiological findings. This peculiar occurrence could be related to the anatomical pathway of the left brachiocephalic vein (BCV). Unlike its right counterpart, the left BCV, which carries contrast agents introduced via the left arm, runs anteriorly between the aortic arch and the sternum before draining into the SVC. This anatomical positioning makes the left BCV more prone to compression between the sternum and the aortic arch or its branches, particularly when the patient is lying in a supine position [6]. Additionally, the left BCV follows a more extended route within the venous system. This longer path may lead to dispersion of the flow and an increase in retrograde flow into the paravertebral venous plexus [6].

In the cases of our patients, a consistent pattern was observed in multiple CT scans: pseudopathologic enhancement consistently appeared at identical locations along the spine. This enhancement was not random. Our hypothesis is that pseudopathologic enhancement is likely to develop in specific areas of the spine, particularly those drained by veins that might have compromised structural integrity. Such veins, being weakened or otherwise altered, are more prone to retrograde flow and vertebral venous congestion.

Traditionally, the vertebral venous plexus is understood to consist entirely of thin-walled and valveless veins, which permit bidirectional blood flow [7,8]. However, a recent anatomical study involving 12 adult cadavers revealed the presence of valves in the posterior external venous plexus, oriented toward the internal vertebral venous plexus [9]. Additionally, it was noted that the walls of the internal vertebral venous plexus are complex and muscular [9]. This finding suggests a more intricate venous structure than previously thought. Nonetheless, further research and histological evidence are necessary to substantiate these findings fully and understand their implications for venous flow dynamics and pathological processes in the spine.

While the pseudopathologic enhancement of thoracic vertebrae is relatively rare, it can occur in patients who do not have thoracic venous obstructions. This phenomenon is particularly notable when contrast agents are administered through the left arm. The importance of recognizing this pattern cannot be overstated, especially in the context of interpreting oncology-related CT scans. Misinterpretation of these enhancements as pathological can lead to unnecessary interventions, such as unwarranted biopsies, or may result in an inaccurate assessment of disease progression, which could significantly impact patient management and treatment decisions.

## Data Availability

Data are contained within the article. No new data were created or analyzed in this study.
